# Migrant perinatal depression study: a prospective cohort study of perinatal depression on the Thai-Myanmar border

**DOI:** 10.1136/bmjopen-2017-017129

**Published:** 2018-01-05

**Authors:** Gracia Fellmeth, Emma H Plugge, Verena Carrara, Mina Fazel, May May Oo, Yuwapha Phichitphadungtham, Mupawjay Pimanpanarak, Naw Kerry Wai, Oh Mu, Prakaykaew Charunwatthana, François Nosten, Raymond Fitzpatrick, Rose Mcgready

**Affiliations:** 1 Nuffield Department of Population Health, University of Oxford, Oxford, UK; 2 Faculty of Tropical Medicine, Mahidol University, Shoklo Malaria Research Unit, Mahidol-Oxford Tropical Medicine Research Unit, Mae Sot, Thailand; 3 Nuffield Department of Medicine, Centre for Tropical Medicine and Global Health, University of Oxford, Oxford, UK; 4 Department of Psychiatry, University of Oxford, Warneford Hospital, Oxford, UK; 5 Department of Clinical Tropical Medicine, Faculty of Tropical Medicine, Mahidol University, Bangkok, Thailand

**Keywords:** mental health, maternal medicine, preventive medicine, public health

## Abstract

**Purpose:**

Perinatal depression is a significant contributor to maternal morbidity. Migrant women in resource-poor settings may be at increased risk, yet little research has been conducted in low-income and middle-income settings. This prospective cohort study of migrant women on the Thai-Myanmar border aims to establish prevalence of perinatal depression, identify risk factors for perinatal depression and examine associations with infant outcomes.

**Participants:**

Participating women are labour migrants and refugees living on the Thai-Myanmar border. A total of 568 women were recruited in their first trimester of pregnancy and are being followed up to 1-year postpartum.

**Findings to date:**

At baseline, women in our study had a median age of 25 years, the predominant ethnicity was Sgaw Karen (48.9%), agriculture was the main employment sector (39.2%) and educational attainment was low with a median of 4 years of education. In the first trimester of pregnancy, a quarter (25.8%; 95% CI 22.3 to 29.5) of all women were depressed as diagnosed by the *Structured Clinical Interview for the Diagnosis of DSM-IV Disorders*.

**Future plans:**

Follow-up is ongoing and expected to continue until January 2018. The prevalence of depression at later stages of pregnancy and during the first postpartum year will be identified, and associations between depression status and demographic, social, migration-related, medical, obstetric and infant factors will be quantified.

**Trial registration number:**

NCT02790905.

**Strengths and limitations of this study**

- To our knowledge, this is the first prospective study of perinatal depression among migrant women in a low-income setting, and our study contributes significantly to the under-researched field of migrant mental health.
- This study will provide the first quantification of disease burden of perinatal depression and identification of associated factors on the Thai-Myanmar border.
- Findings will enable improved detection of perinatal depression as well as earlier and better management of affected women.
- Interviews were carried out by general clinicians rather than psychiatrists. This shortcoming was the result of an absence of psychiatric expertise in this setting. However, the use of generalists including local healthcare workers is a strength for the long-term sustainability of identifying and managing mental disorders in this population.
- There was a 26.1% loss to follow-up at the third trimester in this highly mobile population. Statistical analyses will be conducted to explore differences between those lost and those who completed the study.

## Introduction

Perinatal depression—a depressive episode occurring during pregnancy or up to 12 months postpartum—is a significant contributor to maternal morbidity.^1 2^ Globally, the burden falls disproportionately on those living in poverty. In high-income countries, the period prevalence of depression has been estimated at 18.4% in pregnancy and 19.2% postnatally.^3^ In low-income and middle-income countries (LMIC), prevalence estimates are estimated at 25.3% in pregnancy and 19.0% postnatally.^2^ Point prevalence estimates from meta-analyses have found rates ranging from 7.4% to 12.8% in individual trimesters of pregnancy and a peak of 12.9% in the third month postpartum.^3 4^ However, these meta-analyses are limited to studies from high-income settings, and comparable estimates from LMIC are lacking.

The consequences of perinatal depression are significant. Affected women are at risk of chronic and recurrent depression, and the ability to work and provide care may be impaired. Depression in pregnancy has been linked to negative health behaviours such as substance misuse and poor uptake of antenatal care.^3^ Infants of depressed mothers are at increased risk of preterm birth, low birth weight, stunting in later childhood and poor neurodevelopmental and behavioural outcomes which may persist into adolescence and affect functional outcomes.^5–7^

Migrant women, whom we define as those who have left their place of origin regardless of circumstances, are at particular risk of perinatal depression.^8 9^ Stressors within their family, occupational and social circumstances—many of which may have contributed to their decision to migrate—continue to impact on migrant populations in their place of settlement.^8 9^ Women who resettle within LMIC are at especially high risk.^10^ However, despite the bulk of global migration flows occurring in low-income and middle-income regions, the evidence on migrant mental health remains heavily skewed towards high-income destinations. A systematic review of perinatal mental disorders among migrant women identified 41 studies, of which 37 were conducted in high-income countries, four in middle-income countries and none in low-income countries.^10^ There is thus an urgent need for improved understanding and detection of perinatal depression in LMIC to enable quantification of the disease burden and effective management of the condition.^2^

The Thai-Myanmar border area is home to an estimated 200 000 labour migrants and 145 000 refugees from Myanmar.^11 12^ The prevalence of perinatal depression within this setting has not previously been examined. A prospective cohort study of pregnant migrant women in this low-income setting was set up with the following objectives: (1) to determine the prevalence of perinatal depression; (2) to identify differences in prevalence at various stages of pregnancy and the postpartum period; (3) to identify demographic, social, medical and obstetric factors associated with perinatal depression; and (4) to examine associations between maternal depression and neurodevelopmental outcomes of infants. In this paper, we describe the design, recruitment and characteristics of the cohort.

## Cohort description

### Setting

**Figure 1:**
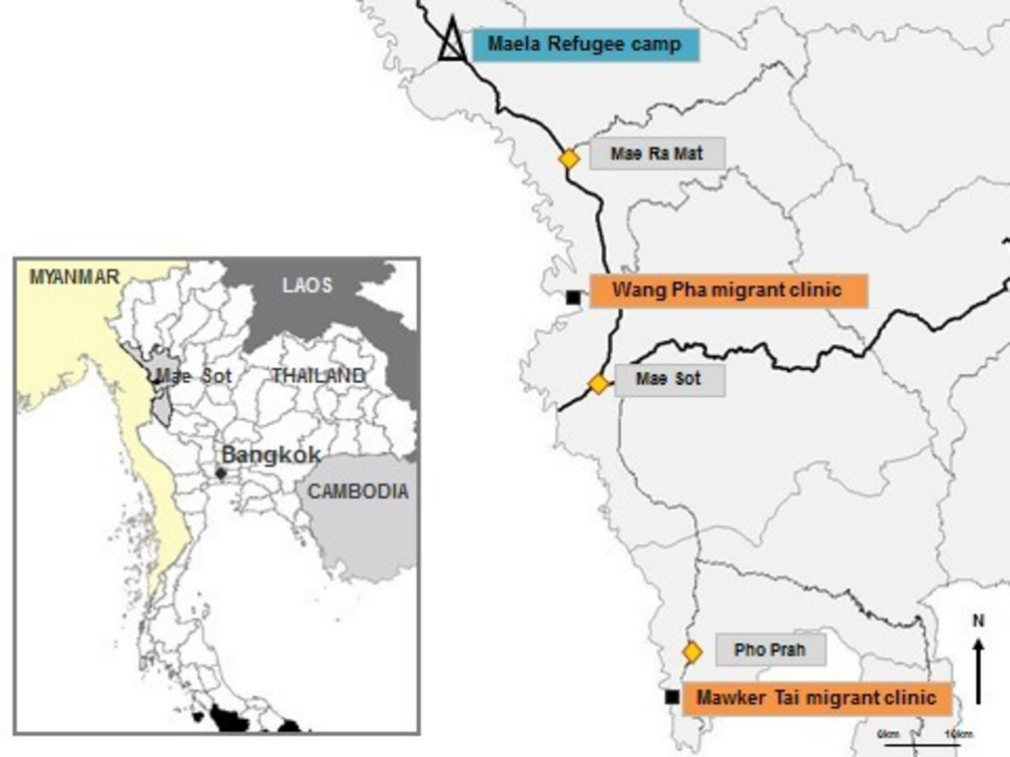
Map of study area showing refugee (∆) and migrant clinics (■) (Credit: Dr Verena Carrara, Shoklo Malaria Research Unit).

The study was carried out at Shoklo Malaria Research Unit (SMRU) in Mae Sot, Tak Province, Thailand. SMRU is a field station of the Mahidol-Oxford Tropical Medicine Research Unit, a research collaboration between Mahidol University (Thailand) and the University of Oxford (UK). SMRU has carried out research and provided maternity services on the Thai-Myanmar border area since 1986. Its clinics are located along the Thai side of the border, 30–60 km north and south of Mae Sot. Care is provided to refugee women and infants in Maela camp (MLA) and to rural labour migrants at Mawker Tai (MKT) and Wang Pha (WPA) (figure 1).

### Population

Refugees live in camps on the Thai side of the border. MLA is the largest refugee camp with a population of 37 000.^13^ Within the refugee camp, non-governmental organisations provide healthcare, education, food rations and housing, and the United Nations High Commissioner for Refugees manages repatriation and resettlement programmes.^13^ Although the refugee camps provide a degree of security, opportunities for work and freedom of movement are severely limited. By contrast, labour migrants are a highly mobile population, residing in rural villages on both sides of the border and often many making daily commutes across the border for work. Labour migrants in this setting work predominantly in the agricultural sector and are paid minimal daily wages.^11 12^ Many labour migrants lack official documentation rendering them vulnerable to fines, arrest and deportation by the Thai authorities, and excluding them from accessing healthcare, social care and education.^11 12^ In this paper, we use the term ‘migrant’ to include both refugee and labour migrant populations.

### Eligibility and recruitment

This prospective cohort study includes first trimester pregnant migrant women attending SMRU antenatal clinics (ANC) at MLA, MKT and WPA. Women were eligible if they were aged 18 years or over, their estimated gestational age (EGA) as determined by ultrasound dating scan was less than 14 weeks, they had a viable pregnancy, planned to deliver at SMRU and were willing and able to participate. Eligible women were approached by a member of the study team while waiting to be seen at ANC. Recruitment took place between October 2015 and April 2016. Follow-up assessments will take place regularly until 12 months postpartum and will be complete in January 2018. Further follow-up is subject to funding.

### Ethics and consent

At recruitment, study staff provided eligible women with verbal and written explanations of the study. It was explained that participation was voluntary, that non-participation would not affect care and that consent could be withdrawn at any time. Women who agreed to participate provided consent in the form of a signature or thumbprint for those with low literacy. Participants are offered a small gift (of approximate value £1) at each visit and any travel costs incurred are reimbursed.

### Instruments

Depression status is being ascertained using the depression items of the *Structured Clinical Interview for the Diagnosis of DSM-IV Disorders* (SCID), a widely used, semistructured diagnostic tool.^14^ The SCID was translated into Burmese and Sgaw Karen by two SMRU clinicians fluent in Burmese, Karen and English. Back-translation was carried out by two further SMRU clinicians who had not seen the original English version. Original and back-translated English versions were compared to ensure that semantic equivalence had been maintained. DSM-IV criteria were applied to SCID responses to establish diagnoses of Major Depressive Disorder, Minor Depressive Disorder and Depressive Disorder Not Otherwise Specified (NOS). The diagnostic category of Depressive Disorder NOS was included to capture the substantial proportion of women with symptoms of depression that were clinically significant but which did not meet the DSM-IV criteria for major or minor depression.

At inclusion in trimester one (T1), the *Refugee Health Screener-15* (RHS-15) was also administered. The RHS-15 screens for psychological and somatic symptoms of depression, anxiety and post-traumatic stress disorder.^15^ The RHS-15 consists of 14 Likert-type response items and a distress thermometer that asks respondents to rate their distress on a visual scale of 1 to 10. Burmese and Sgaw Karen versions of the RHS-15 were acquired from the RHS-15 authors.^16^

Data on demographic, social and migration data were collected using questionnaires. Medical and obstetric data will be obtained from participants’ computerised medical records following delivery. Infant measurements including length and weight are being conducted using standardised instruments. Infant development is being assessed using the Shoklo Developmental Test, a locally developed neurological examination designed for field use in resource-constrained settings.^17 18^ The Shoklo Developmental Test has good correlation with the Griffiths Developmental Scales and has been used in our setting to evaluate the neurodevelopment of infants born to children with malaria in pregnancy and in ongoing studies of neonatal jaundice.^17 19–22^ Mothers’ developing relationships with their infants is being assessed with the Mother-to-Infant Bonding Scale.^23^

### Procedure

A study timeline is shown in table 1. Data are being collected at eight time points: in the first (T1), second (T2) and third (T3) trimesters of pregnancy and at 1 (T4), 3 (T5), 6 (T6), 9 (T7) and 12 (T8) months postpartum. Questionnaires and interviews are conducted by study staff in a private room in Sgaw Karen or Burmese according to women’s preference. Verbal administration (rather than self-completion) is used due to low literacy rates within this population and limited comprehension of health-related written information, even among those able to read.^24^ SCID responses are independently scored by the study physician and an independent physician. Disagreements are resolved by discussion with a psychiatrist (MF). Women with depression are offered counselling and, when appropriate, antidepressant medication and follow-up at SMRU. Women with severe symptoms or active suicidal ideation are admitted for treatment and observation.

**Table 1:** Timeline of data collection

Time		Depression		Demographic and social	Medical factors	Obstetric factors	Infant factors	Infant bonding
		SCID	RHS-15*					
Pregnancy								
T1	First trimester (EGA<14)	X	Full	X				
T2	Second trimester (EGA 18–26)	X	DT					
T3	Third trimester (EGA 28–38)	X	DT	X				
Postpartum								
T4	One month postpartum	X	DT		X	X	X	X
T5	Three months postpartum						X	
T6	Six months postpartum	X	DT				X	X
T7	Nine months postpartum						X	
T8	Twelve months postpartum	X	DT		X		X	X

*At T1, the full RHS-15 was administered. At subsequent visits, only the distress thermometer (DT) component of the RHS-15 was administered.

EGA, estimated gestational age; RHS-15, Refugee Health Screener-15; SCID, Structured Clinical Interview for the Diagnosis of DSM-IV Disorders.

### Quality assurance and control

The study team consists of SMRU physicians, midwives and counsellors. Midwives and counsellors are fluent in Burmese, Sgaw Karen and English and are themselves members of the local migrant community, and therefore sensitive to the needs of the population. Prior to recruitment, the study lead (GF) received training from the American Psychiatry Association in conducting SCID interviews. Counsellors and midwives underwent training in conducting interviews and counselling methods prior to the study. During the first month, all questionnaires and interviews were conducted with the study lead until counsellors and midwives were able to perform them unassisted. Thereafter, the study lead co-conducted interviews at one site per day to ensure quality.

### Sample size

All women attending SMRU ANC in their first trimester of pregnancy during the recruitment period were invited to participate. Based on previous studies in this setting, we assumed a high participation rate and approximately 15% loss to follow-up.^25^ Our target sample size of 500 was based on an assumed approximate overall depression prevalence of 20% and 80% power (with two-sided 95% CIs) to detect associations of approximately 2.5-fold in magnitude and to run multiple regression analyses with up to four independent variables.^26^

### Data security and management

All data are de-identified and entered into a password-protected Microsoft Excel database accessible only to SMRU study staff. Source questionnaires are held securely at SMRU ANC sites. Once follow-up is complete they will be stored at the SMRU head office.

## Findings to date

**Figure 2:**
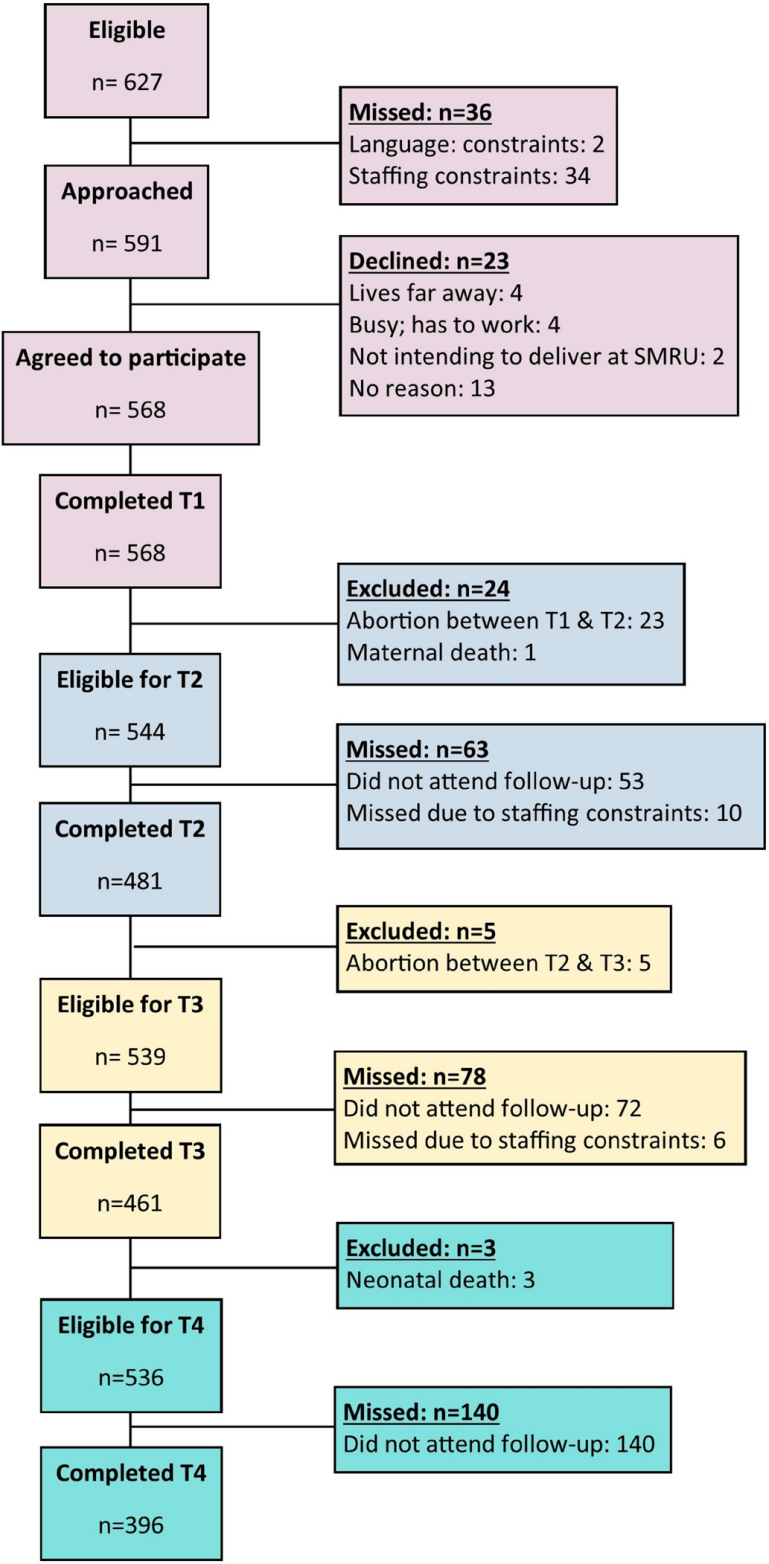
Flow of participants through study from recruitment to T3. SMRU, Shoklo Malaria Research Unit.

Between October 2015 and April 2016, 627 eligible women attended SMRU ANC. Of these, 591 were approached and 568 (90.6% of all eligible; 96.1% of those approached) women agreed to participate. Figure 2 shows the flow of participants through the study from recruitment (T1) through to T3. Follow-up for T4 through T8 is still ongoing. Women who were eligible but missed due to language or staffing constraints did not differ significantly from those included by age, ethnicity or educational level. Of the 568 women who completed T1, 84.7% completed T2% and 81.2% completed T3. Some women who did not attend at T2 returned at T3. The most common reason for participants not returning for follow-up was abortion in early pregnancy.

### Demographic characteristics

At enrolment, the median age was 25 years (table 2) and mean EGA (SD) was 9.6 (2.3) weeks. Sgaw Karen was the predominant ethnicity and language among refugees, while Burman ethnicity and Burmese language were predominant among labour migrant women. The median years of education was 4 years, and almost half (45.4%) of all participants had attended school for under 3 years. The main employment sector was agricultural work (39.2%), although over a third of participants were not in paid employment (35.7%).

**Table 2:** Demographic characteristics of study participants at T1 (n=568)

	All (n=568)	Missing	Labour migrant sites			Refugee camp	
			MKT (n=163)	WPA (n=155)	P value* MKT versus WPA	MLA (n=250)	P value* Ref versus mig
Demographic							
Age, med (range)	25 (18–50)	0	25 (18–45)	26 (18–44)	0.50	25 (18–50)	0.98
**Ethnicity, n (%)**							
Burman	161 (28.4)	0	77 (47.2)	82 (52.9)	0.21	2 (0.8)	**<0.01**
Sgaw Karen	278 (48.9)		61 (37.4)	41 (26.5)		176 (70.4)	
Poe Karen	66 (11.6)		19 (11.7)	24 (15.5)		23 (9.2)	
Burman Muslim	44 (7.8)		0 (0)	1 (0.7)		43 (17.2)	
Other	19 (3.4)		6 (3.7)	7 (4.5)		6 (2.4)	
**Religion, n (%)**							
Buddhist	408 (71.8)	0	152 (93.2)	147 (94.8)	0.40	109 (43.6)	
Christian	115 (20.3)		11 (6.8)	7 (4.5)		97 (38.8)	
Muslim	45 (7.9)		0 (0)	1 (0.7)		44 (17.6)	**<0.01**
**Marital status, n (%)**							
Married/cohabiting	566 (99.6)	0	163 (100.0)	154 (99.3)	0.49	249 (99.6)	1.00
Education and language							
Years of education, med (range)	4 (0–18)	78	3 (0–12)	4 (0–15)	0.42	5 (0–18)	0.02
**Years of education, n (%)**							
Under 3 years, n (%)	255 (45.4)	6	100 (61.7)	60 (39.0)	**<0.01**	95 (38.6)	**<0.01**
3 to 6 years, n (%)	164 (29.2)		35 (21.6)	61 (39.6)		68 (27.6)	
7 to 10 years, n (%)	119 (21.2)		26 (16.1)	23 (14.9)		70 (28.5)	
Over 10 years, n (%)	24 (4.3)		1 (0.6)	10 (6.5)		13 (5.3)	
**Type of school, n (%)**							
Myanmar school	245 (53.4)	116	66 (53.2)	89 (76.7)	**<0.01**	90 (41.1)	**<0.01**
Thai school	9 (2.0)		7 (5.7)	0 (0)		2 (0.9)	
NGO/faith-based school	89 (19.4)		9 (7.3)	3 (2.6)		77 (35.2)	
None	116 (25.3)		42 (33.9)	24 (20.7)		50 (22.8)	
Literate (self-report), n (%)	392 (69.0)	0	98 (60.1)	106 (68.4)	0.13	188 (75.2)	**<0.01**
**Interview language, n (%)**							
Burmese	239 (42.1)	0	91 (55.8)	97 (62.6)	0.05	51 (20.4)	<0.01
Sgaw Karen	280 (49.3)		64 (39.3)	42 (27.1)		174 (69.6)	
Poe Karen	43 (7.6)		7 (4.3)	13 (8.4)		23 (9.2)	
Other	6 (1.1)		1 (0.6)	3 (1.9)		2 (0.8)	
**Languages spoken, n (%)**							
One language	211 (46.2)	82	53 (42.7)	58 (50.4)	0.48	100 (45.9)	0.99
Two to three languages	192 (42.0)		56 (45.2)	44 (38.3)		92 (42.2)	
Four or more languages	54 (11.8)		15 (12.1)	13 (11.3)		26 (11.9)	
Economic							
**Employment sector, n (%)**							
Agriculture	212 (39.2)	2	119 (73.9)	76 (56.3)	**0.02**	17 (6.9)	**<0.01**
NGO	59 (10.9)		2 (1.2)	5 (3.7)		52 (21.2)	
Selling	50 (9.2)		12 (7.5)	17 (12.6)		21 (8.6)	
Other	27 (5.0)		3 (1.9)	6 (4.4)		18 (7.4)	
Housework	193 (35.7)		25 (15.5)	31 (23.0)		137 (55.9)	
Household size, med (range)	4 (1–14)	4	3 (1–14)	4 (2–12)	0.51	5 (1–13)	**<0.01**
Telephone ownership, n (%)	329 (57.9)	0	87 (53.4)	71 (45.8)	0.18	171 (68.4)	**<0.01**
Lifestyle							
**Substance use, n (%)**							
Alcohol	25 (4.4)	0	1 (0.6)	18 (11.6)	**<0.01**	6 (2.4)	**0.04**
Smoking	56 (9.9)	0	17 (10.4)	10 (6.5)	0.20	29 (11.6)	0.22
Chewing tobacco	27 (4.8)	0	23 (14.1)	0 (0)	**<0.01**	4 (1.6)	**<0.01**
Chewing betel	251 (44.2)	0	53 (32.5)	66 (42.6)	0.06	132 (52.8)	**<0.01**
Obstetric							
Parity, med (range)	1 (0–8)	113	1 (0–5)	1 (0–8)	0.43	1 (0–7)	**<0.01**
Planned pregnancy, n (%)	310 (68.3)	114	85 (68.0)	71 (64.0)	0.51	154 (70.6)	0.30
Psychosocial							
History of depression, n (%)	147 (26.0)	2	2 (1.23)	25 (16.3)	**<0.01**	120 (48.0)	**<0.01**
Migration							
**Country now living, n (%)**							
Myanmar	113 (24.5)	108	36 (28.8)	74 (63.2)	**<0.01**	3 (1.4)	**<0.01**
Thailand	348 (75.5)		89 (71.2)	43 (36.8)		216 (98.6)	
**Years in current location**							
Median (range)	9 (1–39)	272	3 (1–33)	10 (1–39)	**<0.01**	10 (1–30)	**<0.01**
≤1 year, n (%)	64 (31.7)		36 (42.4)	19 (29.7)	0.11	9 (17.0)	**<0.01**
≤5 years, n (%)	158 (53.4)		74 (60.2)	41 (47.7)	0.07	43 (49.4)	0.38

*P values calculated using two-group t-tests for continuous data, χ^2^ tests for categorical data and Fisher’s exact test for categorical data with cell counts<5.

MKT, Mawker Tai; MLA, Maela camp; NGO, non-governmental organisation; WPA, Wang Pha.

### Depression status

At baseline, the overall prevalence of depression as diagnosed by the SCID was 25.8% (table 3). There were significant differences in crude prevalence rates of depression between MKT and WPA, and between the migrant sites (MKT and WPA) combined and MLA. Explanations for these differences will be explored through regression analyses.

**Table 3:** First trimester depression status among study participants by site and by migrant status

	All (n=568)	Labour migrant sites			Refugee camp	
		MKT (n=163)	WPA (n=155)	p Value MKT versus WPA	MLA (n=250)	p Value Ref versus mig
Any depression Major depression Minor depression Depression NOS Negative	146 (25.8) 9 (1.6) 37 (6.5) 100 (17.6) 421 (74.2)	29 (17.9) 0 (0) 6 (3.7) 23 (14.2) 133 (82.1)	46 (29.7) 2 (1.3) 19 (12.3) 25 (16.1) 109 (70.3)	0.01	71 (28.4) 7 (2.8) 12 (4.8) 52 (20.8) 179 (71.6)	0.03

MKT, Mawker Tai; MLA, Maela camp; NOS, Not Otherwise Specified; WPA, Wang Pha.

## Strengths and limitations

To our knowledge, this is the first prospective study of perinatal depression among migrant women in a resource-constrained setting. The active screening for depression will inform the early detection and treatment of this condition, enabling affected women to be supported and appropriate interventions to be developed. An improved understanding of the prevalence and risk factors of depression is a cornerstone to addressing the disease burden. Mental disorders are a neglected field in this and other low-income settings. The limited number of previous studies from this region have focused on specific subgroups including refugee children,^27^ Burmese political dissidents living in Bangkok,^28^ labour migrants workers in Mae Sot^29^ and Karenni refugees in northern Thailand.^30^ None have included pregnant or postpartum women. To our knowledge, this is also the first study to include both labour migrants and refugees, enabling direct comparison between these two distinct subgroups of the migrant population. As well as allowing the progression of depression through pregnancy and the postpartum period to be assessed, a significant strength of our cohort design is the collection of data on an extensive array of potential risk and associated factors including demographic, social, medical, obstetric and infant factors. Overall, our study contributes to the under-researched field of migrant mental health from LMIC settings.^10 27^

A further strength of our study is that while most studies of mental disorders use screening tools to make mental state assessments, we used a diagnostic interview tool.^10^ Interviews were conducted by local healthcare staff who are themselves part of the local community. This enabled trust to be established with patients, and ensured high levels of cultural sensitivity. The fact that over 90% of women in this area attend ANC, coupled with our high response rate, means that our sample is representative of the general migrant population.^25^ The inclusion of the category of Depression NOS sheds light on an under-reported group of women who experience symptoms of depression that are clinically significant but do not quite meet the criteria for major or minor depression. In order to increase comparability to findings from other settings, our main statistical analyses will be limited to the more commonly reported categories of minor and major depression. We will also conduct additional analyses to explore the effects of including the NOS group.

There are also a number of limitations to our study. The absence of mental health expertise in our setting meant that it was not possible to obtain specialist psychiatry input.^31 32^ However, by providing experienced local healthcare workers with training in conducting interviews and counselling skills we maximised accuracy as much as possible. Furthermore, in a resource-constrained setting it is more appropriate in the long term for common mental disorders such as depression to be identified and managed by trained local workers, as specialist mental health professionals are rarely available. By training frontline healthcare staff to conduct these assessments and provide counselling, we ensured that our study promoted local capacity building, ownership, scalability and sustainability.^33^

A further limitation is that face-to-face administration of interviews may have resulted in a social desirability bias and a lower willingness to disclose sensitive information.^34^ Given the sensitive nature of many of the issues discussed, including depression, suicidal ideation and behaviour, intimate partner violence and trauma history, participants may have felt uncomfortable discussing these and under-reported relevant experiences, especially during a vulnerable period such as pregnancy. However, there is a strong oral tradition among the local population, and informal discussions are common and well accepted.^35^ We also believe that the sensitivity and local knowledge of the study staff helped to ensure that participants felt comfortable disclosing personal information.

The repeated administration of the SCID may have affected how women responded. We saw no evidence of questionnaire fatigue, perhaps because women in our setting attend clinic on a fortnightly basis through much of their pregnancy, and thus completing the SCID once per trimester was not perceived as burdensome. However, the repeated SCID interviews may have had a therapeutic effect by enabling participants an opportunity to talk and share any worries. This possibility will be taken into account in the interpretation of prevalence of depression after the baseline assessment.

Infants’ neurodevelopmental outcomes will need to be interpreted with caution as assessments within the first year of life may not be sensitive enough to identify subtle differences between infants. Ideally, the cohort of infants would be followed up longer term. Nevertheless, it may be possible by 12 months to see trends in the progression of global development. The use of a more widely used tool such as the Bayley Scales would have been preferable. However, staffing and resource constraints, the length of the full Bayley test and a number of test items being difficult to convey in the local cultural context meant this was not possible.^17 18^ The Shoklo Developmental Test has been used extensively in our setting and its strong correlation with the Griffiths Developmental Scale—a standardised neurodevelopmental assessment tool—gives confidence to the results.^17 18^ Should further follow-up become possible, it would be important to consider a wider range of validated instruments to test child outcomes.

Finally, our overall loss to follow-up to date of approximately 20% is higher than our anticipated loss of 15%. This figure may increase in subsequent postpartum waves. We plan to explore differences between those included and lost in our analyses in order to assess potential implications for generalisability of results.

## Conclusion

Addressing perinatal depression among migrant communities in LMIC is necessary to promote maternal mental health and address key sustainable development goals including ensuring good health and well-being, establishing gender equality and reducing global inequalities both within and among countries. Establishing the prevalence of and risk factors for perinatal depression among migrant women on the Thai-Myanmar border will enable the burden of disease to be quantified, and earlier, more effective identification and management of affected women. We expect that observations and recommendations arising from this study will be of importance and relevance to other LMIC settings.
